# Particulate Matter and Volatile Organic Compounds Emissions and Their Health Impact: Spotlight on Solid Fuel Combustion

**DOI:** 10.3390/toxics13020088

**Published:** 2025-01-24

**Authors:** Jian Sun, Meng Wang, Jie Tian

**Affiliations:** 1 Department of Environmental Science and Engineering, Xi’an Jiaotong University, Xi’an 710049, China; 2 Department of Civil and Environmental Engineering, The Hong Kong Polytechnic University, Kowloon, Hong Kong, China; 3 Key Lab of Aerosol Chemistry & Physics, SKLLQG, Institute of Earth Environment, Chinese Academy of Sciences, Xi’an 710061, China; tianjie@ieecas.cn

## 1. Introduction

Solid fuel combustion, while a crucial source of energy in many regions around the world, remains one of the primary contributors to air pollution, with significant implications for human health [1,2]. The burning of solid fuels—ranging from biomass to coal—releases a variety of particulate matter (PM) and volatile organic compounds (VOCs) into the atmosphere [3,4]. These emissions, which vary depending on fuel type and combustion conditions, can pose a serious threat to public health, contributing to respiratory diseases [5], cardiovascular conditions, and potentially even neurological and reproductive dysfunctions [6,7]. Understanding the complex relationship between these emissions and health outcomes remains an ongoing challenge, especially given the diversity of combustion sources and the various chemical and physical pollutants involved.

This Special Issue, Particulate Matter and Volatile Organic Compounds Emissions and Their Health Impact: Spotlight on Solid Fuel Combustion, aims to advance our understanding of these critical issues. Through the publication of seven original research papers, this Special Issue provides fresh insights into the health effects of solid fuel combustion and explores gaps in the current literature [8,9]. It pays particular attention to the chemical profiles, health risks, and mechanisms of toxicity of particulate and gaseous emissions [10]. These contributions not only deepen our understanding of the adverse impacts of solid fuel burning on human health but also pave the way for future research that can guide policy and mitigation strategies.

## 2. Overview of the Papers in This Special Issue

The first paper, Sources and Specified Health Risks of 12 PM_2.5_-Bound Metals in a Typical Air-Polluted City in Northern China during the 13th Five-Year Plan (contribution 1), highlights coal combustion as a major source of PM_2.5_-bound metals (22.3%) and identifies it as a significant contributor to health risks, particularly concerning carcinogenic metals like Cr and As. The seasonal variation in emissions, with higher levels in winter, correlates with increased use of solid fuels during colder months in China, further emphasizing the role of solid fuel combustion in urban air pollution.

Following this, the second paper (contribution 2), Unveiling the Aftermath: Exploring Residue Profiles of Insecticides, Herbicides, and Fungicides in Rice Straw, Soils, and Air Post-Mixed Pesticide-Contaminated Biomass Burning, explores the broader environmental consequences of solid fuel combustion. The authors focus on biomass burning from pesticide-contaminated rice straw, providing an in-depth analysis of the residue profiles in air, soil, and plant matter. This study highlights the additional layers of complexity introduced by the presence of agricultural chemicals in biomass combustion, showing that these compounds can significantly alter the toxicity of the emitted particulate matter and VOCs.

The third paper, Chemical Source Profiles and Toxicity Assessment of Urban Fugitive Dust PM_2.5_ in Guanzhong Plain, China (contribution 3), shifts the focus to urban environments, where fugitive dust emissions from solid fuel combustion are a growing concern. It identifies biomass burning as a key contributor to road dust in Baoji, directly linking solid fuel combustion to atmospheric dust and PM_2.5_. The enrichment of elements like Zn and Se further suggests that industrial systems, which often rely on solid fuel combustion for energy, play a significant role in dust pollution in the region. This highlights the need for effective control measures to reduce both industrial and residential emissions from solid fuels, which can exacerbate air pollution and health risks.

In the fourth paper, Adverse Effects of Prenatal Exposure to Oxidized Black Carbon Particles on the Reproductive System of Male Mice (contribution 4), the authors investigate the reproductive toxicity of oxidized black carbon particles, a major component of PM from biomass burning. This study provides compelling evidence of the adverse effects of prenatal exposure to these particles, showing a potential link between air pollution from solid fuel combustion and reproductive health outcomes, particularly in male offspring.

The fifth paper, Comprehensive Assessment of Pollution Sources and Health Impacts in Suburban Area of Shanghai, focuses on a suburban region in China (contribution 5), discusses how rapid industrialization and the continued use of solid fuels contribute to significant air pollution. The study emphasizes industrial sources and their contribution to respiratory health risks, implying that emissions from solid fuel combustion, especially in nearby industries, may be a major factor in the observed health impacts in suburban Shanghai.

The sixth paper, Long-Term Observation of Mixing States and Sources of Vanadium-Containing Single Particles from 2020 to 2021 in Guangzhou, China (contribution 6), provides a long-term observational study of vanadium-containing particles, which are often emitted from ships. The study’s findings that V-containing particles are mixed with sulfate and nitrate indicate that solid fuel combustion sources (including on ships) play a role in the formation of secondary particulate matter, particularly in urban areas impacted by transportation and industrial emissions. Additionally, the reduction in V-containing particles following the clean fuel policy may reflect efforts to reduce emissions from both marine and industrial solid fuel combustion sources, highlighting the connection between solid fuel use, ship emissions, and air quality improvements.

The seventh and final paper, Gas Particle Partitioning of PAHs Emissions from Typical Solid Fuel Combustions as Well as Their Health Risk Assessment in Rural Guanzhong Plain, China (contribution 7), investigates the partitioning behavior of polycyclic aromatic hydrocarbons (PAHs) during the combustion of solid fuels. The study assesses the associated health risks, particularly in rural areas where such fuels are commonly used. The findings underscore the importance of understanding the chemical transformations of pollutants during combustion, as well as their distribution between gas and particle phases, to fully assess their health impacts.

## 3. Scientific Meaning of This Special Issue

One of the main contributions of this Special Issue is its focus on the complex nature of pollutants released from solid fuel combustion. While the general health impacts of particulate matter and VOCs are well documented, the chemical composition of these emissions and their toxicity vary significantly depending on the type of fuel used, combustion conditions, and geographical context. The papers in this Special Issue address these gaps by providing detailed chemical profiles, toxicity assessments, and long-term observations that offer new insights into the specific pollutants that contribute to health risks.

Additionally, the papers highlight the role of secondary pollutants—such as oxidized black carbon and PAHs—that form during combustion and their often-underappreciated toxicity. While many studies focus primarily on primary emissions, these secondary compounds can be equally or even more harmful, necessitating a deeper examination of their formation and impacts.

This Special Issue also explores the intersection between environmental pollution and chemical contaminants, such as pesticides in agricultural residues, which are often overlooked in studies of solid fuel emissions. This broader perspective is essential for understanding the full range of pollutants involved in biomass burning and their potential synergistic effects on health.

As the sources of air pollutants are not completely independent, their interactions should be considered, especially those from solid fuel combustion. The fugitive dust in the outback and ship emissions in coastal areas both showed variations in mixing state and/or toxicity, indicating the crucial role of atmospheric aging on primary PM. This finding shed light on the fact that focusing on interactions among varied emission sources, particularly solid fuel combustion sources which exist widely, potentially leads to new research orientations for source-dependent air pollutants and the associated toxicity.

## 4. Future Directions in Research

Despite the advances made by the studies in this Special Issue, significant knowledge gaps remain that need to be addressed in future research. Several key areas deserve particular attention:

### 4.1. Health Effect Mechanisms

While much is known about the associations between particulate matter and various health conditions, the underlying biological mechanisms have not been fully understood to date, especially for VOCs. Future research should focus on elucidating the molecular and cellular pathways through VOCs exerting harmful effects, particularly in vulnerable populations such as pregnant women, children, and the elderly.

### 4.2. Long-Term Health Outcomes

Many of the studies in this Special Issue focus on short-term exposure to solid fuel combustion pollutants. Longitudinal studies are needed to assess the long-term health outcomes of chronic exposure, particularly for non-communicable diseases such as cardiovascular diseases, cancers, and neurodegenerative disorders.

### 4.3. Impact of Combustion Variability

The diversity of solid fuels used worldwide means that the emissions from combustion are highly variable. Future research should explore the differences in emissions from different types of biomass (e.g., rice straw, wood, animal dung) and coal, considering both combustion technologies and environmental conditions. This will allow for more tailored mitigation strategies based on local fuel use and pollution profiles.

### 4.4. Exposure Assessment and Mitigation

Given the continued use of solid fuels in many parts of the world, particularly in low-income regions, there is a pressing need for research focused on exposure assessment and effective mitigation strategies. These include both technological innovations to improve combustion efficiency and policy interventions to reduce the use of high-emission fuels.

### 4.5. Epidemiological Studies

Considering that solid fuel combustion scenarios often occur in rural areas, epidemiological studies on the topic of household fuel combustion or air pollution and human health, although discussed in a considerable number of papers, are far from enough to emphasize the impact of solid fuel combustion sources on human health. This calls for more research on the epidemiology of solid fuel combustion sources, which is more important than ever.

### 4.6. Interdisciplinary Approaches

The complexity of the health impacts of solid fuel combustion requires interdisciplinary research that combines environmental science, toxicology, epidemiology, and social sciences. Future studies should adopt a holistic approach, integrating data from different disciplines to better understand the full scope of the risks associated with these emissions.

## 5. Conclusions

The research presented in this Special Issue contributes significantly to the growing body of knowledge on the health impacts of particulate matter and VOCs emitted from solid fuel combustion (contribution 2). By addressing critical gaps in understanding—ranging from chemical profiles and toxicity to health risk assessments—these papers set the stage for future research that will ultimately guide public health strategies and policy decisions aimed at mitigating the adverse effects of air pollution. The interactions between solid fuel combustion and other sources, or the aging effects of solid fuel combustion depending on circumstance, should be focused on to open up a new field of solid fuel combustion studies. Continued efforts to explore the mechanisms of toxicity, long-term health outcomes, and strategies for reducing emissions will be essential for improving public health and mitigating the risks associated with solid fuel use.

As we look to the future, it is clear that a multidisciplinary, global approach is necessary to address the health challenges posed by solid fuel combustion. The work presented here is an important step in this direction, and we hope that it will inspire further research that continues to uncover solutions to this pressing global health issue.
